# Loss of the extracellular matrix glycoprotein EMILIN1 accelerates Δ16HER2-driven breast cancer initiation in mice

**DOI:** 10.1038/s41523-023-00608-0

**Published:** 2024-01-06

**Authors:** Andrea Favero, Ilenia Segatto, Alessandra Capuano, Maria Chiara Mattevi, Gian Luca Rampioni Vinciguerra, Lorena Musco, Sara D’Andrea, Alessandra Dall’Acqua, Chiara Gava, Tiziana Perin, Samuele Massarut, Cristina Marchini, Gustavo Baldassarre, Paola Spessotto, Barbara Belletti

**Affiliations:** 1 Unit of Molecular Oncology, Centro di Riferimento Oncologico (CRO) di Aviano, IRCCS, National Cancer Institute, 33081 Aviano, Italy; 2 Faculty of Medicine and Psychology, Department of Clinical and Molecular Medicine, University of Rome “Sapienza”, Santo Andrea Hospital, 00189 Rome, Italy; 3 Medical Department, University of Udine, Udine, Italy; 4 Unit of Pathology, Centro di Riferimento Oncologico (CRO) di Aviano, IRCCS, National Cancer Institute, 33081 Aviano, Italy; 5 Unit of Breast Surgery, Centro di Riferimento Oncologico (CRO) di Aviano, IRCCS, National Cancer Institute, 33081 Aviano, Italy; 6 School of Biosciences and Veterinary Medicine, Biology Division, University of Camerino, via Gentile III da Varano, 62032 Camerino, Italy

**Keywords:** Breast cancer, Cancer microenvironment, Oncogenes

## Abstract

The extracellular matrix (ECM) is an important component of the tumor microenvironment and undergoes extensive remodeling during both initiation and progression of breast cancer (BC). EMILIN1 is an ECM glycoprotein, whose function has been linked to cancer and metastasis. However, EMILIN1 role during mammary gland and BC development has never been investigated. In silico and molecular analyses of human samples from normal mammary gland and BC showed that EMILIN1 expression was lower in tumors than in healthy mammary tissue and it predicted poor prognosis, particularly in HER2-positive BC. HER2+ BC accounts for 15-20% of all invasive BC and is characterized by high aggressiveness and poor prognosis. The Δ16HER2 isoform, a splice variant with very high oncogenic potential, is frequently expressed in HER2+ BC and correlates with metastatic disease. To elucidate the role of EMILIN1 in BC, we analyzed the phenotype of MMTV-Δ16HER2 transgenic mice, developing spontaneous multifocal mammary adenocarcinomas, crossed with EMILIN1 knock-out (KO) animals. We observed that Δ16HER2/EMILIN1 KO female mice exhibited an accelerated normal mammary gland development and a significantly anticipated appearance of palpable tumors (13.32 *vs* 15.28 weeks). This accelerated tumor initiation was corroborated by an increased number of tumor foci observed in mammary glands from Δ16HER2/EMILIN1 KO mice compared to the wild-type counterpart. Altogether our results underscore the centrality of ECM in the process of BC initiation and point to a role for EMILIN1 during normal mammary gland development and in protecting from HER2-driven breast tumorigenesis.

## Introduction

Breast Cancer (BC) is the most common cause of malignancy-related death in women worldwide. It is a highly heterogeneous pathology and its survival rates have improved thanks to early diagnosis and increased number of available and effective therapies^1,2^. Human epidermal growth factor receptor 2 (HER2) is a tyrosine kinase receptor overexpressed in approximately 20% of all invasive BC, contributing to high aggressiveness and poor prognosis. In the literature, three naturally occurring splice variants of HER2 are described, namely p100, Herstatin, and Δ16HER2^3^. In particular, Δ16HER2, expressed in all HER2+ human BC, is formed following the excision of exon 16, resulting in the loss of a small juxtamembrane region of the receptor and the gain of the ability to form constitutively active homodimers. This triggers sustained downstream signaling, preferentially transduced by Src kinase^4,5^. Δ16HER2 is co-expressed with full-length HER2 in human BC and is reported to significantly impact on aggressiveness and response to therapies^6,7^. Collectively, Δ16HER2 isoform can be considered a driver of human HER2-positive BC.

The tumor microenvironment (TME) is a highly heterogeneous component of the tumor that includes fibroblasts, endothelial cells, adipocytes, immune and inflammatory cells and a non-cellular three-dimensional constituent, known as extracellular matrix (ECM)^8^. The ECM is composed by macromolecules that provide structural and biochemical support to surrounding cells, acting also as a source of signaling molecules^8^. Although much less studied than other TME components, it is well established that an altered ECM composition and deposition can affect cell transformation, tumor dormancy and awakening and also, cancer progression, *via* direct action on tumoral cells and *via* dysregulation of the stromal counterpart^8–10^. ECM is constantly deposited, remodeled, and degraded to maintain tissue homeostasis, even under physiological conditions, but in cancer it is extensively reorganized and acquires different composition and stiffness^11^. Among ECM proteins, the elastin microfibrillar interface protein-1 (EMILIN1) is a member of the EMI Domain ENdowed (EDEN) superfamily, whose structure comprises five different domains: the N-terminal domain, called EMI domain; the central coiled-coil region; the leucine zipper domain; the collagenous domain; and the C-terminal gC1q domain. The gC1q is where the residue E933 resides, necessary to mediate the interaction between EMILIN1 and the α4β1 and α9β1 integrins^12,13^. EMILIN1 is closely associated with elastic fibers, known to be involved in skin homeostasis and carcinogenesis, affecting on tumor cell proliferation and lymph node invasion^14^. The study of the EMILIN1 KO mouse model reinforced those notions, suggesting a protective role of EMILIN1 in tumor growth and, possibly, in the metastatic spread to lymph nodes^14,15^.

Here, by using genetically modified animal models, we investigate the possibility that loss of EMILIN1 expression impacts on mammary gland development and Δ16HER2-driven tumorigenesis, shedding new light onto the involvement of EMILIN1 in the early steps of Δ16HER2 mammary epithelial cell transformation and tumor formation.

## Results

### EMILIN1 is expressed in normal murine and human mammary gland and downregulated in human breast cancer

**Fig. 1:**
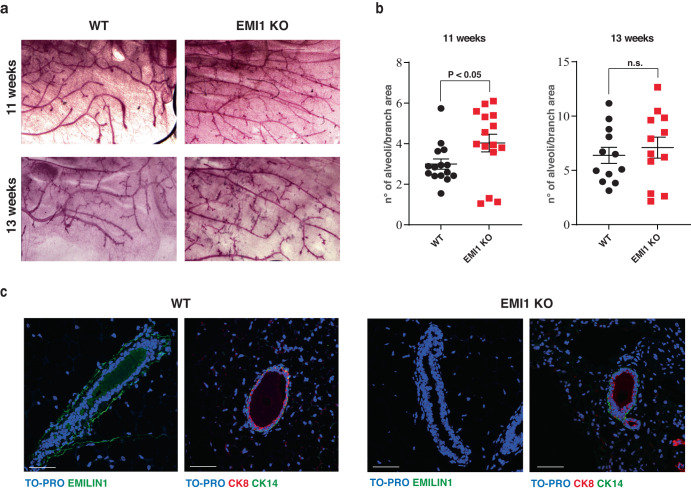
In mice, EMILIN1 is localized in the extracellular matrix that surrounds the ductal tree of the mammary gland and the tumor mass. **a** Whole mount of mammary glands collected from WT (left) and EMILIN1 KO (right) female mice at different stages of development. At least four mice/stage/genotype were evaluated. **b** Quantification of ductal branching in terms of secondary branching and alveoli formation per each primary branch. At least three branches/mammary gland/mouse of 11W and 13W females were analyzed. Graphs report the mean ± SEM. Statistical significance was calculated using Student *t*-test. **c** Immunofluorescence analysis of mammary ducts from WT (left) and EMI1 KO (right) showing the deposition of EMILIN1 in the extracellular matrix of the ductal tree (top) and the localization of cytokeratin 8 (luminal marker, red) and cytokeratin 14 (basal marker, green), at 13 W of age. At least three mice/genotype were evaluated. Scale bar 50 µm.

EMILIN1 expression and role in mammary gland function and development are not known. Therefore, we initially analyzed EMILIN1 deposition in normal murine mammary gland (MMG) and whether its ablation had an impact on MMG structure and function. To this end, we collected MMG samples from both WT and EMILIN1 (EMI1) KO female mice at 11 weeks of age (11W) and 13 weeks of age (13W). We observed an increase in ductal sprouting in EMI1 KO MMG at 11W, partially maintained at 13W, in line with the fact that EMI1 KO female mice are fertile and perfectly fit in nursing their litter (Fig. 1a, b).

**Fig. 2:**
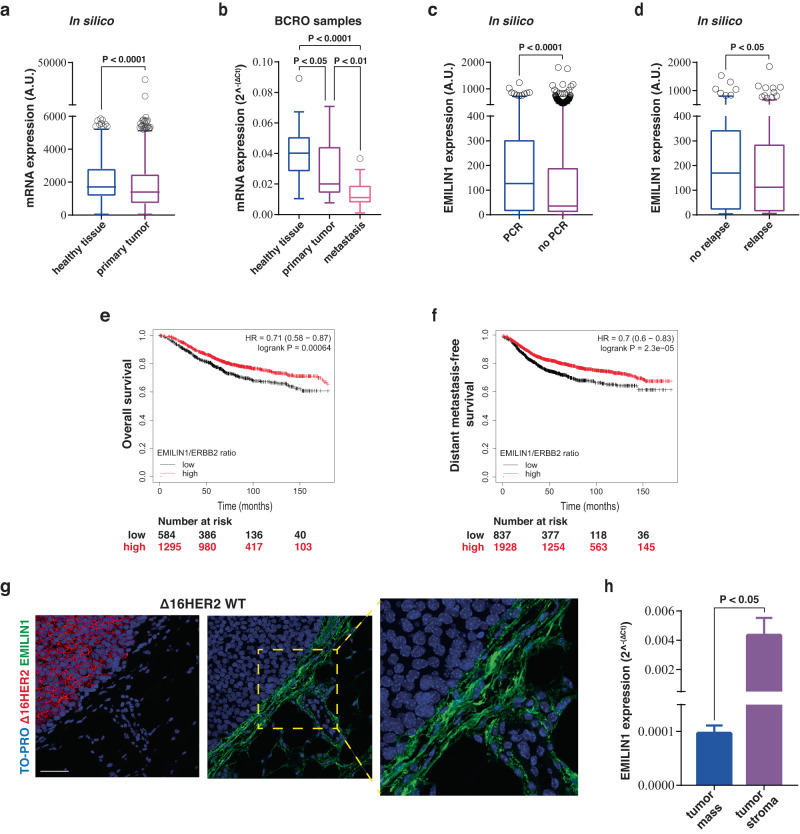
EMILIN1 is expressed in human mammary gland and downregulated in breast cancer. **a** Box plot represents EMILIN1 expression in human breast cancer. Data were retrieved from TNMplot, which collects RNA-seq data from TCGA-BRCA database and TARGET projects, comparing healthy tissues (*n* = 403) and primary mammary tumors (*n* = 1097). **b** Box plot reports EMILIN1 expression in a panel of BC samples collected in our Institute (BCRO samples; healthy tissues, *n* = 20; primary tumors, *n* = 46; metastasis, *n* = 20). **c**, **d** Graphs report the median gene expression of EMILIN1 in patients evaluated for their pathological complete response (PCR) to neoadjuvant chemotherapy (**e**) and in patients grouped for the relapse-free survival at 5 years (**f**). Data were collected from ROC plotter. In all box plots, boxes include first and third quartile, line indicates the median and whiskers indicate the minimum and maximum data values. Outliers are reported at the extremities. Statistical significance was calculated with Mann–Whitney test (**a**, **c**, **d**), or Kruskal–Wallis test (**b**) as more appropriate. **e**, **f** Overall survival (**e**) and distant metastasis-free survival (**f**) Kaplan–Meier plots of patients divided by high and low EMILIN1/ERBB2 ratio. The analysis was performed with online Kaplan–Meier Plotter (KMplot), taking into account all subtypes of breast cancer, and the statistical evaluation was performed directly by KM plotter, using the logrank test. **g** Representative pictures of ∆16HER2 (left, red) and EMILIN1 (right, green) localization in the tumor microenvironment of Δ16HER2 mice. **h** qRT-PCR analysis of murine EMILIN1 transcript levels inside the tumor mass and in the tumor stroma. At least four tumor masses of two mice were analyzed. Graph reports the mean±SEM. Statistical significance was calculated using Student *t*-test and indicated by a *P* < 0.05.

Based on immunofluorescent staining, we observed that EMI1 was expressed in the MMG and was deposited in the extracellular matrix (ECM) surrounding and sustaining the mammary ducts (Fig. 1c). Given this specific localization, which could suggest an involvement in the formation and/or maintenance of MMG architecture, we asked whether its loss or alteration could impinge on mammary tumorigenesis. We first interrogated the TCGA-BRCA database to evaluate EMILIN1 expression and observed that it was significantly downregulated in tumors compared to healthy mammary tissue (Fig. 2a). However, the variability in the stromal tissue content between tumors and healthy mammary tissues introduced an unpredictable factor in these results. Therefore, we tested EMILIN1 expression across a large panel of primary and metastatic BC specimens collected in our Institute. These samples have highly variable but, as a whole, more comparable stromal levels. Further we used surrounding mammary tissues as controls. Again, not only EMI1 expression was lower in tumors than in healthy tissues, as seen in silico, but it progressively diminished as BC progressed to a metastatic stage (Fig. 2b). This finding prompted us to verify if EMILIN1 could have a predictive value, at clinical level. A high EMILIN1 expression correlated with pathological complete response (PCR) to neoadjuvant chemotherapy (Fig. 2c) and with relapse-free survival at 5 years (Fig. 2d). Interestingly, a high EMI1/HER2 ratio correlated with better distant metastasis-free survival and overall survival of BC patients, while patients characterized by low EMILIN1 and high HER2 expression displayed a worse prognosis (Fig. 2e, f). Together, these data suggested that loss of EMI1 may play a role in BC.

### In FVB MMTV Δ16HER2 mice, EMILIN1 is deposited in the ECM surrounding the tumors mice

Given the above data, we decided to focus on the HER2+BC subtype and took advantage of the FVB Δ16HER2 transgenic mouse model, expressing the Δ16 variant of HER2 under the mouse mammary tumor virus (MMTV) promoter, in which females spontaneously develop very aggressive multifocal and invasive mammary carcinomas, with onset of palpable tumors at an average age of 15.28 weeks^16–18^. We intercrossed Δ16HER2 male mice with EMI1 KO females^19^ and generated the FVB MMTV-Δ16HER2/EMILIN1 knock-out (Δ16HER2/EMI1 KO) mouse colony. First, we looked at the expression of EMI1 in MMG bearing tumors and observed that it was deposited in the ECM surrounding the tumor mass, while it was not present inside the tumor (Fig. 2g). Consistently, when tumor masses were separated from the surrounding stroma, EMILIN1 transcription was detected essentially in the stromal compartment while the tumor component expressed only negligible levels (Fig. 2h). These results indicated that mammary carcinoma cells were not the main source of EMI1 in this context and suggested that stromal fibroblasts might be the ones responsible for EMI1 production and deposition around the tumor, as previously shown in other contexts^15^.

### Loss of EMILIN1 accelerates mammary gland development in Δ16HER2 mice

**Fig. 3:**
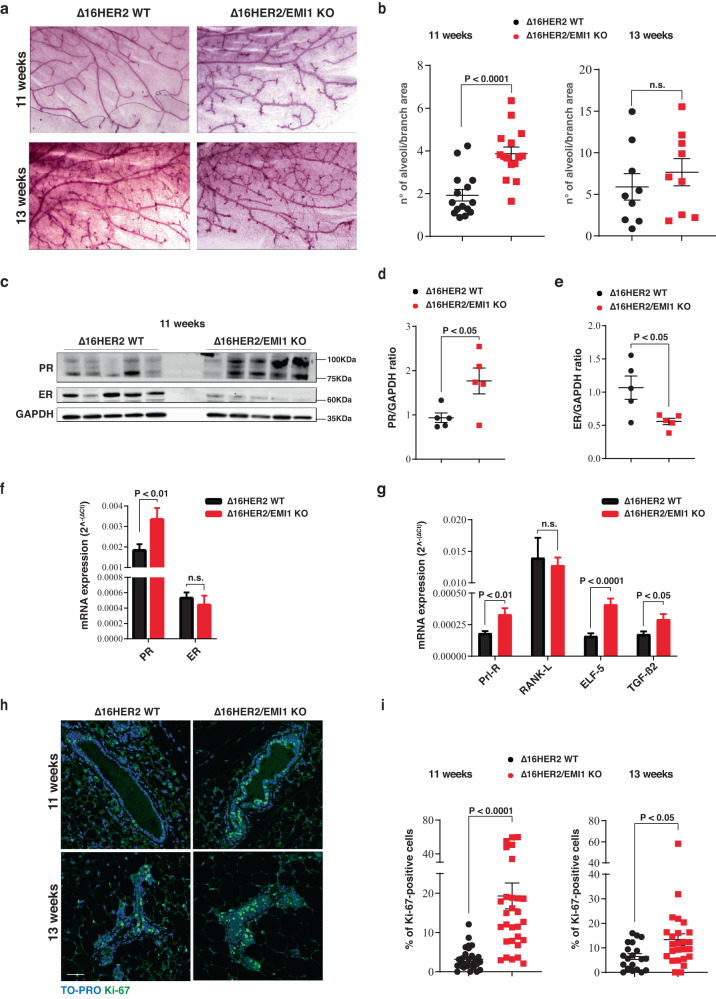
EMILIN1 regulates mammary gland development and proliferation, in mice. **a** Whole mount of murine mammary glands collected from ∆16HER2 WT (left) and ∆16HER2/EMI1 KO (right) animals at the indicated time-points. At least four mice/stage/genotype were analyzed. **b** Quantification of ductal branching in terms of secondary branching and alveoli formation per each primary branch. At least three mammary gland/mouse of 11 W were analyzed. **c** Representative western blot analysis of indicated proteins in lysates from ∆16HER2 WT and ∆16HER2/EMI1 KO mammary glands at 11 W. Five animals/genotype were evaluated. **d**, **e** Graphs report the quantification of estrogen receptor (ER) (**d**) and progesterone receptor (PR) (**e**) protein level displayed in the western blot in (**c**). **f** qRT-PCR analysis of PR and ER transcript levels in ∆16HER2 WT and ∆16HER2/EMI1 KO mammary glands of 11 W mice. **g** qRT-PCR evaluation of indicated transcripts in ∆16HER2 WT and ∆16HER2/EMI1 KO mammary glands of 11 W mice. For each qRT-PCR, mammary glands of at least four mice/genotype were analyzed. **h** Representative immunofluorescences of ∆16HER2 WT (left) and ∆16HER2/EMI1 KO (right) animals at 11 W (top) and 13 W (bottom) showing cell proliferation indicated by the Ki-67 marker (green) in the mouse mammary gland. Scale bar 50 µm. **i** Graphs represent quantification of the Ki-67 immunofluorescent analysis at 11 W (left) and 13 W (right), normalized on the total number of cells per field. At least five fields/mammary gland/mice were evaluated. All graphs report the mean ± SEM. Statistical significance was calculated using Student *t*-test and indicated by a *P* < 0.05.

Next, we investigated the impact of EMI1 loss at different stages of Δ16HER2-driven tumorigenesis. First, we collected MMG from Δ16HER2/EMI1 WT and KO virgin females at pre-tumoral stages, specifically 11 W and 13 W (n. 5/genotype/stage). When examining the MMG architecture, we observed that Δ16HER2/EMI1 KO mice displayed a significantly higher degree of side branching and initial processes of alveologenesis. This difference was robust and consistent at 11W and less pronounced at 13W (Fig. 3a, b), as previously seen in EMI KO female mice (Fig. 1a, b).

It is known that hormonal levels are tightly regulated during MMG development, playing distinct and fundamental roles in the different stages of branching and alveologenesis^20^. Estrogen levels primarily regulate the initial steps of development, specifically ductal elongation and bifurcation, while progesterone becomes more active during the subsequent steps of side branching and alveologenesis, together with prolactin, which is also the main regulator of the lactogenic differentiation^21^. We thus looked at the status of activation of these hormonal pathways in 11 W MMG tissue and observed that Δ16HER2/EMI1 KO mice displayed definitely lower levels of estrogen receptor (ER) at protein level and significantly higher of progesterone receptor (PR), both at protein (Fig. 3c–e) and RNA level (Fig. 3f), compared to Δ16HER2/EMI1 WT. We also examined other known regulators of secondary branching, such as prolactin receptor (Prl-R), Elf5, Transforming Growth Factor (TGF)-β2 and RANK-L^22–24^. The results confirmed the trend observed for ER and PR and further suggested that, at 11 W of age, different transcriptional programs were in place in the MMG of Δ16HER2/EMI1 WT and KO mice (Fig. 3g). Finally, immunofluorescence analysis of the proliferation marker Ki-67 demonstrated a consistent and significant increase in proliferation in MMG from Δ16HER2/EMI1 KO compared to WT, particularly evident at 11 W but maintained at 13 W (Fig. 3h, i).

Together, these data indicated that, while ductal elongation/bifurcation stage was still ongoing in 11 W MMG Δ16HER2/EMI1 WT female animals, the 11 W MMG from Δ16HER2/EMI1 KO mice were already progressing to the subsequent stage, in which estrogen had already carried out its function and was therefore downmodulated, while progesterone and prolactin were driving the side branching of the mammary acini.

So, in the context of Δ16HER2 MMG, the KO of EMI1 induced an acceleration of the MMG development.

### EMILIN1 ablation accelerates Δ16HER2-driven tumor initiation

**Fig. 4:**
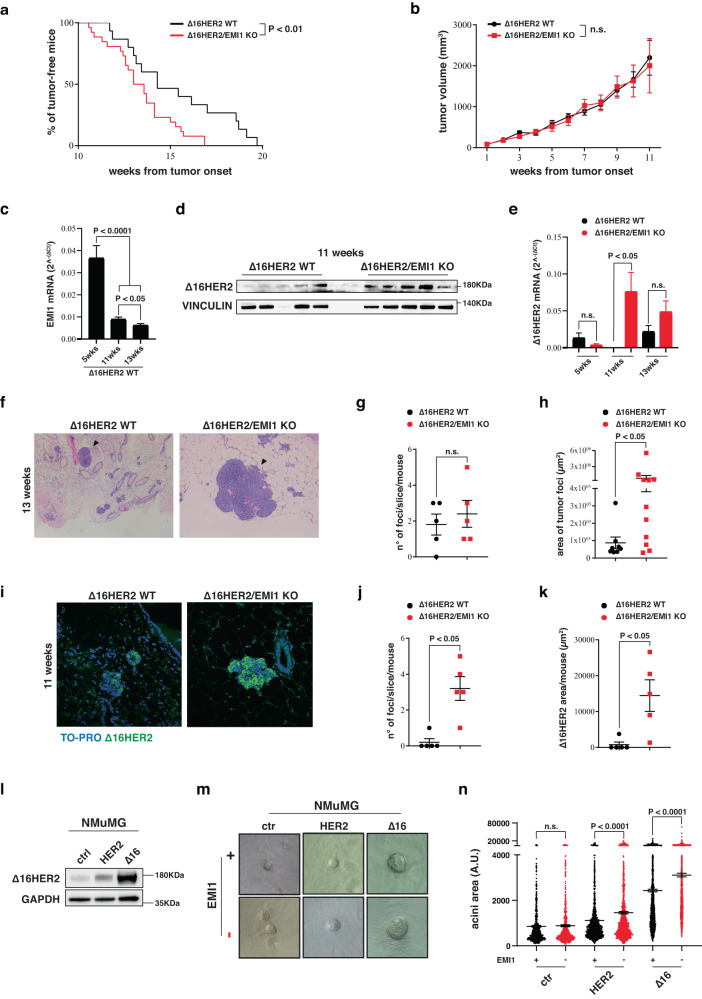
EMILIN1 loss induces an acceleration of the tumor onset in a model of ∆16HER2-driven tumorigenesis. **a**, **b** Graphs report the onset (**a**) and growth (**b**) of palpable tumors derived from ∆16HER2 WT (black line) and ∆16HER2/EMI1 KO (red line) mice. At least 15 animals per genotype were evaluated. **c** qRT-PCR analysis of EMILIN1 transcript levels in ∆16HER2 WT mammary glands of 5 W, 11 W and 13 W mice. At least three mice/genotype were analyzed. **d** Immunoblotting analysis of indicated protein levels in 11 W ∆16HER2 WT and ∆16HER2/EMI1 KO MMG. Five different mice/genotype were evaluated. **e** qRT-PCR analysis of ∆16HER2 splicing variant in ∆16HER2 WT and ∆16HER2/EMI1 KO MMG from 5 W, 11 W and 13 W mice. At least three mice/genotype were analyzed. **f** Hematoxylin and eosin staining of mammary gland slices from 13 W ∆16HER2 WT (left) and ∆16HER2/EMI1 KO (right) animals depicting the onset of spontaneous tumor foci. **g**, **h** Graphs represent the number (**g**) and the area (**h**) of tumor foci detected in 13 W ∆16HER2 WT and ∆16HER2/EMI1 KO mice. At least five mice per genotype were analyzed. **i** Immunofluorescence of MMG from 11 W ∆16HER2 WT (left) and ∆16HER2/EMI1 KO (right) mice representing the onset of tumor foci based on the staining for ∆16HER2 (green). Scale bar 50 µm. **j**, **k** Graphs report the number (**j**) and the area (**k**) of tumor foci/slice/mouse detected in 11 W ∆16HER2 WT and ∆16HER2/EMI1 KO animals. **l** Western blot analysis of indicated protein levels in NMuMG cells transfected with the indicated transgene/splicing variant. **m** Representative pictures of the 3D culture of NMuMG cells stimulated with the recombinant gC1q domain of EMI1 (+) or PBS (−) as control. **n** Graph reports the quantification of acini area measured in the three-dimensional culture assay. Three replicates were analyzed and a total of at least six-hundred acini were measured for each condition. All graphs report the mean ± SEM. Statistical significance was calculated using Student *t*-test and indicated by a *P* < 0.05.

We wondered whether the hormone receptor alterations and the accelerated mammary gland development that we observed in the pre-neoplastic Δ16HER2/EMI1 KO MMG could also have an impact on tumorigenesis. We thus evaluated the appearance of palpable tumors and observed that Δ16HER2/EMI1 KO female mice exhibited a significantly earlier tumor onset compared to the WT counterpart (13.32 vs 15.28 weeks) (Fig. 4a). This anticipation was noteworthy, especially when considering the inherently aggressive nature of Δ16HER2-driven tumorigenesis. We then followed tumor growth over a period of approximately 3 months after the appearance of palpable tumors, measuring tumor masses once a week. However, no significant difference was observed in the growth of tumors between the two cohorts (Fig. 4b), suggesting that, once appeared, tumors grew at a very comparable manner. Accordingly, the difference observed in anticipated tumor onset tended to disappear at 20 weeks of age and the two genetic backgrounds became very similar in terms of tumor multiplicity and weight of the tumor masses (Supplementary Fig. 1a–c). These findings suggested that EMI1 was not able to interfere with Δ16HER2-driven proliferation. This possibility was also confirmed by the observation that EMILIN1 RNA levels progressively decreased in the weeks preceding tumor onset (Fig. 4c), suggesting that its expression needed to be reduced to facilitate the tumor initiation process. Additionally, Δ16HER2 was readily expressed as early as 11 W in EMI KO mammary glands, both at RNA and protein level, aligning with the earlier tumor onset observed in this genetic background (Fig. 4d, e). Expression levels of α4β1, α9β1 integrins and TGFβ, on the other hand, remained similar between the two genotypes and were relatively stable over time (Supplementary Fig. 2).

We thus directed our focus toward the early stages of tumorigenesis. To this aim, we collected MMG from Δ16HER2/EMI1 WT and KO mice at 13 W, a stage when KO mice already exhibited palpable tumors, and 11 W, when only microscopic-level tumor foci could be detected. Hematoxylin and Eosin (H&E) staining data hinted at a higher number of tumor foci in 13 W Δ16HER2/EMI1 KO MMG, although the difference was not statistically significant. However, these foci displayed notably larger areas when compared to the WT counterpart (Fig. 4f–h). We next assessed the presence of small tumor foci through immunofluorescent staining of Δ16HER2 and confirmed that, already at 11 W, Δ16HER2/EMI1 KO group of mice displayed a significantly higher number and larger size of tumor foci in comparison to the Δ16HER2/EMI1 WT group (Fig. 4i–k).

These results were further corroborated in vitro using the NMuMG (Normal Murine Mammary Gland) cell line, transfected to overexpress either the Δ16HER2 or the HER2 full length (HER2 fl) (Fig. 4l). We challenged these cells to grow in a 3D-Matrigel environment and form colonies in the presence or absence of EMI1 in the culture medium (Fig. 4m, n). Both HER2 fl and Δ16HER2 increased the ability of NMuMG cells to form 3D mammary acini, and both types of HER2 transformed cells grew in larger colonies in the absence of EMI1 in the media, simulating the EMI KO context. As expected by the enhanced transforming potential of the HER2 spliced form, Δ16HER2 transformed NMuMG cells formed larger colonies compared to the HER2 fl ones (Fig. 4m, n).

As EMILIN1 deposition is known to exhibit a distinctive association with elastic fibers, we sought to determine whether its absence could impact the stiffness of the mammary ECM, as reported in various other tissues^25,26^. Our aim was to understand whether EMILIN1 loss influenced this aspect of the mammary microenvironment and whether the earlier tumor onset seen in Δ16HER2/EMI1 KO mice could, in part, be attributed to altered ECM stiffness. To investigate this, we assessed the deposition of elastic fibers in 11 W MMG using Masson’s trichrome staining (Supplementary Fig. 3a). We quantified the amount of elastic fibers deposited in at least five ducts *per* MMG and calculated the ratio between the area covered by the collagens (blue staining) around the mammary ducts and the total area of the ducts for each mammary gland. However, our analysis did not reveal any significant differences between the two genotypes (Supplementary Fig. 3b).

Collectively, these findings suggest that the absence of EMILIN1 provides an advantage for Δ16HER2-driven mammary tumorigenesis initiation, and this advantage is not associated with any noticeable alteration in the deposition of elastic fibers.

### EMILIN1 loss impacts on mammary gland adipocytes size and lipid metabolism

**Fig. 5:**
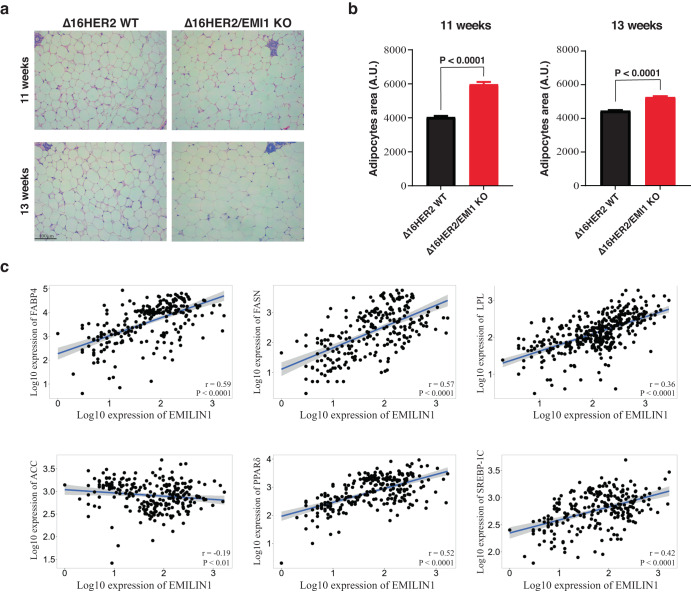
EMILIN1 loss affects lipid metabolism and size of mammary gland adipocytes. **a** Hematoxylin and eosin pictures of mammary gland adipocytes of 11 W (top) and 13 W (bottom) mice. Five mice/genotype were analyzed. Both the third and the fourth mammary glands were evaluated and at least five fields for each slide were analyzed. **b** Graphs report the dimension of mammary gland adipocytes in mice of 11W (left) and 13 W (right) obtained from the hematoxylin and eosin sections. Graphs report the mean ± SEM. Statistical significance was calculated using Student *t*-test and indicated by a *P* < 0.05. **c** Spearman’s correlation between EMILIN1 and the indicated genes involved in fatty acid metabolism. The analysis and the statistical tests were performed using the TNMplot tool for gene *vs* gene correlation in human normal breast.

From macroscopic observations during necroscopy, we consistently noted that Δ16HER2/EMI1 KO mice exhibited a greater amount of fat in their MMG. Given that obesity is a widely recognized risk factor for the development of breast cancer, as well as several other cancer types^27^, we decided to better characterize this phenotype and its potential implication for tumorigenesis. Although the growth of both genotypes, with or without Δ16HER2, from 4 W to 13 W did not highlight any significant difference in weight (data not shown), we conducted an analysis of the adipose tissue by measuring the adipocyte area in both the third and fourth MMG (Fig. 5a, b). Δ16HER2/EMI1 KO displayed larger adipocytes, particularly at 11 W but maintained also at 13 W, which might be in line with the anticipated tumor onset that we observed. However, when we examined several key components of lipid metabolism and fatty acid synthesis, we were surprised to find that most of them were expressed at lower levels in (Supplementary Fig. 4a). No differences were detected in the glucose metabolism either (Supplementary Fig. 4b, c). These findings suggest that a higher extracellular availability of lipids may be present in Δ16HER2/EMI1 KO MMG, potentially explaining the lower expression of factors in the fatty acid synthesis pathway. This possibility was indirectly supported by in silico correlation analyses in human normal mammary tissue, revealing a positive correlation between EMILIN1 expression and most of the genes involved in lipid metabolism (Fig. 5c).

### A functional integrin binding domain is necessary for EMILIN1 to rescue the anticipated mammary tumor onset in Δ16HER2/EMI1 KO mice

The anticipated tumor onset observed in Δ16HER2/EMI1 KO animals made us wonder whether the phenotype was caused by the absence of the EMI domain, the gC1q domain or both. To investigate this aspect, we took advantage of the EMILIN1-E933A (E1-E933A) transgenic mouse model that was generated and previously characterized in our laboratory^28^. These mice express a human EMILIN1 carrying the E933A mutation, which abolishes the ability to bind α4β1 and α9β1 integrins. Tissues from this mouse model were already tested for the expression of E1-E933A, showing that transcriptional levels were very similar to the ones found in WT animals^28^. TGF-β1 protein levels, whose maturation from pro-TGF-β1 can be regulated by the N -terminal EMI domain, were also maintained at similar level in WT and E1-E933A animals^29^. To evaluate whether the binding to integrins, contained in gC1q domain, was important for EMILIN1 function in Δ16HER2-related context, we crossed the E1-E933A mice with the Δ16HER2/EMI1 KO ones and generated the new Δ16HER2/EMI1 KO/E1-E933A mouse colony (Δ16HER2/EMI1 KOtg).

**Fig. 6:**
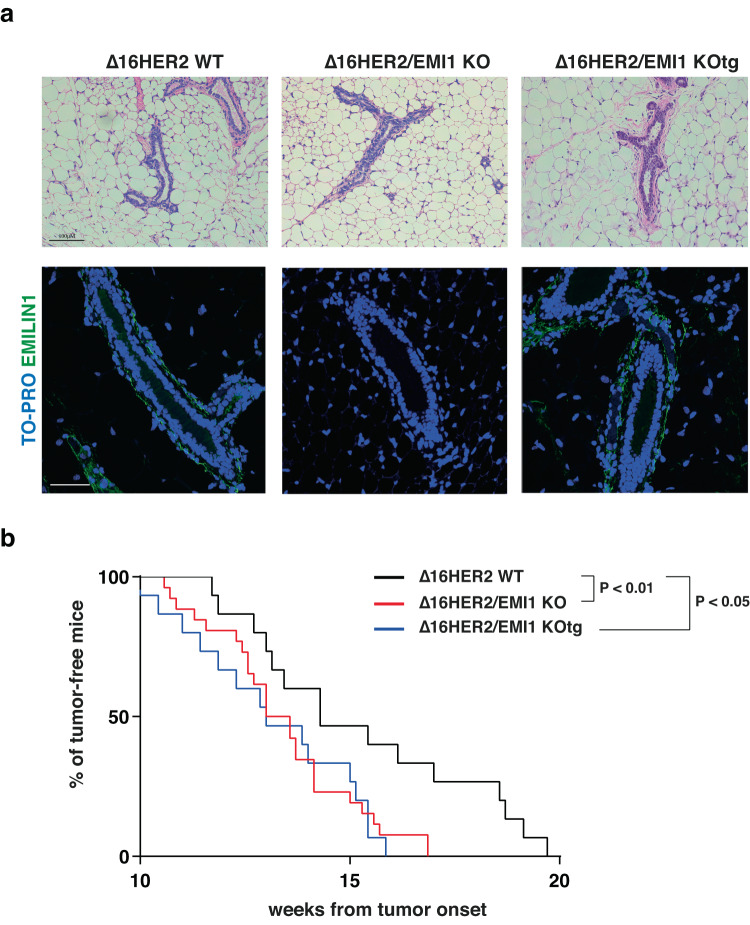
A functional integrin binding domain is necessary to prevent early tumor onset in ∆16HER2/EMI1 KO mice. **a** Hematoxylin and eosin pictures (top) and immunofluorescence images of EMILIN1 (green) of mammary glands collected from ∆16HER2 WT (left), ∆16HER2/EMI1 KO (middle), and ∆16HER2/EMI1 KOtg (right) mice at 11 W of age. At least three mice per genotype were evaluated. TO-PRO was used to stain nuclei in immunofluorescent images. Scale bar 50 µm. **b** Graph report the onset of palpable tumors derived from ∆16HER2 WT (black line), ∆16HER2/EMI1 KO (red line), and ∆16HER2/EMI1 KOtg (blue line) mice. At least 14 animals per genotype were evaluated. In all graphs, significance was calculated by Student *t*-test and indicated by a *P* < 0.05.

Although E1-E933A deposition in the mammary tissue was very similar to the one of EMI1 WT (Fig. 6a), the evaluation of tumor onset by palpation showed that appearance of palpable tumor masses in Δ16HER2/EMI1 KOtg mice was completely overlapping with the one Δ16HER2/EMI1 KO animals (13.27 vs 13.32 weeks) (Fig. 6b), clearly indicating that the gC1q domain and, thus, EMI1 binding to integrins, was a critical event in regulating the tumor onset of Δ16HER2 transgenic mice.

## Discussion

The mammary gland is a dynamic and plastic tissue undergoing dramatic changes throughout its developmental process. Interestingly, many of the pathways responsible for regulating mammary gland development can, when aberrantly activated, lead to the transformation of mammary epithelial cells, ultimately resulting in cancer^30,31^. In both normal development and cancer progression, the extracellular matrix (ECM) plays a pivotal role by maintaining mammary gland structure, influencing mammary cell plasticity and transformation and eventually, BC initiation, growth, and progression.

The role of EMILIN1 in normal mammary gland and in BC has still to be elucidated. Our analyses of mouse mammary glands suggested that while the loss of EMI1 did not yield any physiological or pathological consequences (EMI1 KO mice exhibited a normal lactating phenotype), EMI1 played a crucial role in ensuring the balanced and timely development of the mammary gland. It has been reported that EMILIN1 mRNA level changes based on mammary tumor grade^32^. Yet, very little is known regarding the expression of EMILIN1 in BC, except for recent work of Sharma et al., which identified several unique EMILIN1 transcripts significantly downregulated in various BC subtypes, including luminal, basal, and HER2+ BC, when compared to the Normal-like counterpart in the TCGA dataset^33^. Here, we aimed to bridge this gap and directly analyze *EMILIN1* expression using RNA-seq data from the TCGA-BRCA dataset and a substantial collection of human BC samples from our Institution. Our results are in line with previous data and suggested a tumor suppressive role for EMILIN1 in BC, as already seen in other contexts^14,28,34^. Notably, we observed that EMILIN1 expression was progressively lost as we transitioned from healthy mammary tissue to primary breast cancer. Although this finding could be influenced by variations in stromal content in healthy *versus* tumor samples, we found an even more pronounced loss of EMILIN1 expression in metastatic specimens. Interestingly, EMILIN1 loss of expression was able to predict patient survival in HER2+ BC population, suggesting it may specifically play a role in this BC subtype. This hypothesis is independently supported by the notion that the promoter regions of ITGA4 and ITGA9 genes, the two α-integrins responsible for EMILIN1 cellular binding, are hypermethylated in BC, particularly in HER2+ tumors^35^. These results suggest that HER2 and EMILIN1-α4β1/α9β1 integrins signaling pathways may interact with one another in normal mammary tissue and the biological consequences of their dysregulation are yet to be discovered.

To delve deeper into this issue and characterize the role of EMILIN1 in mammary gland and BC, we employed a transgenic mouse model of HER2-driven mammary tumorigenesis. The HER2+ BC is frequently diagnosed as a high-grade tumor and, together with the overexpression of the amplified HER2 oncogene, is found to co-express the Δ16HER2 splicing variant, to varying extents. This transcript displays the skipping of exon 16 of HER2, which leads to impairment of intramolecular disulfide-bridge formation and promotion of constitutively active homodimers, eventually triggering constitutive oncogenic signaling. Using Δ16HER2 and EMI1 KO mice, we first discovered that ablation of EMILIN1 affected the normal mammary gland architecture, accelerating secondary branching process and alveologenesis, at time points (11 W) at which the wild-type MMG was still undergoing primary branching and ductal elongation steps.

A higher levels of progesterone receptor, leading the secondary branching of the mammary gland, as well as of prolactin receptor, leading the alveologenesis process, possibly explained the anticipated MMG development that we observed in Δ16HER2/EMI1 KO mice at macroscopic level. A relation between ECM molecules, their binding to integrins and estrogen activity in the mammary tissue has been reported in the literature^36,37^. The intriguing possibility EMILIN1 could directly impact on ER expression or stability, in the MMG or using co-culture of mammary epithelial cells and fibroblasts, will certainly deserve further investigation.

Next, we looked at tumor appearance and discovered that ablation of EMI1 expression resulted in an anticipation of tumor onset by 2 weeks (13.32 vs 15.28 weeks). This was particularly significant given the inherently aggressive nature of this tumor model, in which female mice develop multifocal mammary carcinomas at multiple glands with 100% penetrance^16^. Notably, despite this significant acceleration in tumor onset, once tumors emerged, their growth rates in both genotypes were remarkably similar, potentially due to the rapid and aggressive nature of Δ16HER2-driven tumors, which quickly reach a growth plateau, making it difficult to discern differences due to the early onset.

These findings suggest that EMILIN1 may play a role in the initial stages of mammary tumorigenesis but may not significantly affect tumor growth once tumors are established. This is also in line with the observed localization of EMILIN1, deposited in the ECM that surrounded the normal mammary ducts and the tumor masses, while being completely absent within the tumors. The low level of EMILIN1 expression, coupled with the reduced levels of the α4β1/α9β1 integrin receptors on HER2+ cancer cells, reported by others^35^, and the potent activity of the Δ16HER2 oncogene, may explain why we could not detect an antiproliferative effect of EMILIN1 in the tumor mass, while we could observe it in pre-neoplastic mammary glands at 11 W and 13 W.

We next explored potential factors that could be responsible for the observed early tumor initiation phenotype. Notably, Δ16HER2/EMI1 KO mice exhibited a larger amount of fat within the MMG and an increased adipocyte size, a well-established predisposing factor for the onset of breast cancer. However, we did not find a linear correlation with key actors of the lipid metabolism and fatty acid synthesis. However, it is known that an increased fatty acid synthesis does not always relate to obesity and indeed lipid deprivation may increase the activity of different enzymes involved in the fatty acid synthesis^38^. Additionally, extracellular lipid availability can influence de novo lipid synthesis^39^. Therefore, it is possible that lower fatty acid synthesis in Δ16HER2/EMI1 KO mice may be due to a higher extracellular lipid availability, which warrants further investigation. Also, we ruled out the possibility that differences in ECM stiffness in EMI1 KO MMG were responsible for the early tumor initiation, as there were no evident abnormalities in elastic fiber content.

Finally, we turned our attention to discern which domain of EMILIN1 could be responsible for the observed phenotype. The most likely candidates were the N-terminal EMI domain, able to regulate TGF-β maturation, and the C-terminal gC1q domain, responsible for the interaction with the α4β1 and α9β1 integrins. To tackle this question, we established a novel mouse colony through the interbreeding of EMI1 KO-E1-E933A with MMTV-Δ16HER2 mice, thus yielding the Δ16HER2/EMI1 KOtg line. The EMI1 KO-E1-E933A model, previously characterized in our lab, is an EMILIN1 KO mouse in which the mutated gene of human EMILIN1 has been re-inserted and is expressed at comparable levels of the WT counterpart. The E933A mutation impairs the interaction between the gC1q domain and the α4β1 and α9β1 integrins, thus abolishing all functions mediated by this engagement. Our analyses on Δ16HER2/EMI1 KOtg female mice revealed that palpable tumors appeared at the same age as in Δ16HER2/EMI1 KO counterparts, thus clearly indicating that EMI1 interaction with integrins was necessary to protect from the anticipated onset phenotype.

One limitation of our study is that due to the complete absence of nodal metastases in the FVB MMTV-Δ16HER2 mouse model, we could not assess the impact of EMILIN1 loss on the maintenance of lymphatic homeostasis and the regulation of lymph nodal spreading, as previously observed in other cancer types^14,28,40^. Nonetheless, our study of human breast cancer samples revealed a significant decrease in EMILIN1 expression in metastatic lesions, most of which were collected from lymph nodal locations. This finding supports the notion that the EMI1 loss represents a critical step in the process of lymphatic dissemination. Further studies using more appropriate models will be necessary to fully address this issue.

In summary, our data support that EMILIN1 plays a role in controlling mammary gland development and initiating breast cancer in a murine model of HER2+ BC. The precise mechanism behind this tumor initiation phenotype has still to be fully elucidated, but our data indicate a dependency on the interaction between the gC1q domain of EMILIN1 and the α4/α9β1 integrins. This knowledge may also have clinical impact for HER2+ BC patients, since it is well established that deranged integrin signaling may result in increased proliferation and trastuzumab resistance^41^. Moreover, α4β1 engagement has been associated with the homing of endothelial and monocyte precursors to tumors^42^, as well as the adherence of bone marrow-derived cells to tumor-associated endothelium^42,43^.

We expect that a more comprehensive exploration of the mechanism and implications derived from the loss of interaction between EMILIN1 and α4/α9β1 integrins, will provide us with a more profound understanding of the role played by basal membrane disruption in tumor initiation.

## Methods

### Study approval and primary tumor collection

Human specimens were collected from BC patients undergoing surgery in our Institute (BCRO samples), upon signing an informed consent form. The research was permitted by the Institutional Review Board of CRO Aviano (IRB-06-2017) and complied with all relevant ethical regulations, including the Declaration of Helsinki.

BC specimens were immediately frozen and stored at −80°C or formalin fixed, as previously described^44^.

### Cell culture, transfection, and three-dimensional (3D) mammary epithelial cell cultures

NMuMG cells were a kind gift of Dr Andrei V. Bakin (Roswell Park Comprehensive Cancer Center, Buffalo, NY)^45^ and were grown in DMEM (Thermo Fisher Scientific Inc.) supplemented with 10% FBS (Thermo Fisher Scientific Inc.) in standard conditions of 37 °C and 5% CO_2_. NMuMG cells carrying the HER2WT transgene and the Δ16HER2 splicing variant were generated as previously described^18^. Three-dimensional cell culture was performed embedding NMuMG cells (7 × 10^3^ cells) as single cells in Cultrex® RGF-BME, Type 2 Select (RGF-BME, 2%) (Bio-Techne), mixed with the medium and layered on the top of a bottom layer of polymerized RGF-BME (8.5 mg/ml), in a 12-well chamber slide (ibidi). Both top and bottom layers were mixed with recombinant gC1q domain (or PBS, as control) at the final concentration of 20 µg/ml. Recombinant gC1q was produced as previously described^46^. Embedded cells were incubated at 37 °C for 7 days and the acini were measured with the ImageJ software.

### Animal experimentation

Animal experimentation was approved by the Italian Ministry of Health and our Institutional Animal Care and Use Committee (OPBA) (authorizations #616/2015-PR and #630/2020-PR). All the in vivo experiments were conducted strictly following internationally accepted guidelines for animal research (FELASA). Mice colonies were housed in the animal facility of CRO Aviano under controlled environmental parameters (22 °C with 40–60% of humidity), pathogen-free condition, and following a 12-h dark/light cycle. Mice were monitored twice a week for the entire duration of the project and euthanized by injection of excess of anesthetic.

The MMTV-Δ16HER2 EMILIN1 knock-out (EMI1 KO) and the MMTV-Δ16HER2 EMILIN1 knock-out/E933A (EMI1 KOtg) mice were generated by crossing male FVB Δ16HER2^16^ with female FVB EMILIN1 knock-out or with EMILIN1 knock-out carrying the E933A mutant. All genotypes were verified by PCR analysis on DNA extracted from tail biopsies, using the MyTaq^TM^ Extract-PCR Kit (Bioline).

Sequences of the primers used for the genotyping are listed in Supplementary Table 1.

Agarose gel electrophoresis was used to detect PCR products. Gels were prepared using TBE-buffer 1× (Tris 54 Mm, EDTA 0.5 M pH8 20 mM, boric acid 27.5 mM) and added with Ethidium Bromide (Merck).

All mouse samples used for the study were collected post-mortem, during necroscopy.

### Evaluation of tumor onset and progression in transgenic mice

Δ16HER2-bearing mice were monitored by palpation once per week to accurately determine the tumor onset, starting from 8 weeks of age. Tumor progression was evaluated by caliper measurement and tumor mass volume was calculated with the following formula: (Length × Width^2^)/2.

Animals were euthanized at the endpoint of the experiment, at 11, 13 and 20 weeks of age, depending on the tumorigenesis stage examined. At the time of necroscopy tumor masses and mammary glands were collected for the analysis.

### Collection of mouse mammary gland and whole mount staining

Following euthanasia, thoracic and abdominal mammary glands were excised from the skin of virgin female mice at different weeks of age. Immediately after dissection, glands were spread on a glass slide and fixed in 4% paraformaldehyde (PFA) for two hours at room temperature (RT). Once fixed, PFA was eliminated by washing mammary glands in Phosphate Buffered Saline (PBS, Merck) and aqueous solution of 2% Carmine (Merck) and 5% Alum (Merck) was used to stain specifically the epithelial structures. Increasing concentrations of ethanol (EtOH) were then used to dehydrate the mounts (1 h for each concentration). After that, mammary glands were immersed in xylol overnight (ON) at RT to delipidate the mammary fat pad and increase transparency. As last step, mammary glands were mounted with cover-slips using the Eukitt mounting media and analyzed under a stereo microscope (Leica M205 FA).

### Histological and immunofluorescence staining

Mouse thoracic and abdominal mammary glands explanted from 11 and 13 weeks of age where fixed in formalin ON, embedded in paraffin and cut into 2μm-thick sections with a microtome. Gland morphology and neoplastic foci presence were evaluated by Hematoxylin and Eosin (H&E) staining. At least 5 mice/stage/genotype were analyzed. For immunofluorescence (IF) staining, tissue sections were rehydrated in 10 mM citrate buffer pH 6.0, then antigen retrieval was performed (20 min, 550 W). Samples were then left at RT until the cool-down was complete. Permeabilization was performed with 0.2% Triton X-100 (Merck) in PBS for 5 min at RT for HER2 (Abcam). After the permeabilization step, samples were blocked in with 10% normal goat serum in PBS, incubated ON at 4 °C with the primary antibody, then incubated with secondary specific antibodies and TO-PRO-3 (Invitrogen; 1:500). For Ki-67 (Abcam) staining, samples were permeabilized at RT for 10 min with 0.4% Triton X-100 in PBS, then blocked with 10% normal goat serum and incubated ON at RT with the primary antibody. Incubation with secondary antibodies was performed as previously described. Primary antibody used were: HER2 (Abcam, #ab134182, 1:100), Ki-67 (Abcam, #ab15580; 1:200), and EMILIN1 (rabbit polyclonal As556, home-made; 1:200). Secondary antibodies used to perform the experiments were: AlexaFluor® 488- or 568- conjugated (Invitrogen, 1:200). Samples were analyzed with Leica TSC SP8 confocal laser-scanning system. LAS Leica and Volocity (PerkinElmer) software were used to analyze the collected images. For Masson’s trichrome staining, mammary gland slices were dewaxed in deionized water and stained with hematoxylin for 5 min. The samples were then washed with tap water for 5 min and then briefly cleaned with deionized water. After this step, the slices were stained with acid fucsin (Merck) for 5 min and then washed with deionized water. The samples were then immersed in a phosphotungstic acid/phosphomolybdic acid solution (Merck) for 5 min and then stained with aniline blue for 5 min (Merck). After this step the slices were immersed in 1% acetic acid for 2 min, washed with deionized water, and dehydrated with ethanol. Finally, the samples were clarified with xylene and mounted with the Eukitt mounting medium.

### Adipocyte characterization

Adipocyte size was evaluated on sections stained with hematoxylin and eosin (H&E). At least 5 different fields were acquired under a Leica DM750 microscope equipped with a Leica ICC50W camera. The Adiposoft plugin of ImageJ was used to measure the adipocytes dimensions and the results were manually reviewed to assess the correct evaluation by the software.

### RNA extraction and qRT-PCR

RNA extraction from murine mammary glands and human specimens was performed using Trizol reagent (Roche) and homogenizing the specimen grinding the frozen tissue with MACSTM Octo Dissociator MACS (Miltenyi Biotec), as previously described^17,18^. NanoDrop 3300 (Thermo Fisher Scientific Inc.) was used for total RNA spectrophotometric quantification and retro-transcription was achieved by using GoScript™ Reverse Transcription Mix, Random Primers (Promega), in accordance with provider’s instructions. qRT-PCR with 2X SsoFast EvaGreen ready-to-use reaction cocktail SsoFastTM EvaGreen® Supermix, (BioRad) was conducted for target quantification. All primers were purchased from Merck and are listed below. EvaGreen dye incorporation in the PCR products was monitored in real-time using the CFX96 Touch Real-Time PCR Detection System (BioRad). Ct values were normalized over mouse GAPDH or human GAPDH housekeeping gene, as appropriate.

The list of all primers used is provided in Supplementary Table 1.

### Preparation of mammary gland lysates and Western Blot analysis

Total proteins were extracted from whole mammary gland by tissue disruption using the MACS^TM^ Octo Dissociator (Miltenyi Biotec) and subsequently quantified using Bradford protein assay (BioRad). Immunoblotting analyses were performed by separating proteins in 4-20% SDS-PAGE Criterion Precast Gel (BioRad), followed by transfer to nitrocellulose membranes (GE Healthcare). 5% non-fat dry milk in TBS-0.1% Tween20 was used to block membranes and the incubation with primary antibody was performed ON at 4 °C. Primary antibodies used were: HER2 (Abcam, #ab134182; 1:1000), PgR (Thermofisher, PA5-16440; 1:250), ER (Thermofisher, MA1-411; 1:250), GAPDH (Cell Signaling, #5174; 1:1000) and Vinculin (Santa Cruz, sc7649, N19; 1:1000). Membranes were washed in TBS-0.1% Tween20 and incubated at RT for 1 h with the appropriate horseradish peroxidase-conjugated secondary antibodies (GE Healthcare; 1:5000) for ECL detection (Clarity Western ECL Substrate, BioRad). Uncropped scans of blots are displayed as supplementary figures in Supplementary Information.

### Statistical analysis

All the graphs and the statistical analyses were performed using Prism version 7.00 (GraphPad, Inc.). Data were compared using Student’s *t* or Mann–Whitney test, as appropriate, and indicated in each figure. A minimum of three biologically independent samples was used for statistical significance. Differences were considered significant at *P* < 0.05. When not otherwise specified, mean and standard deviation are shown in all graphs. Kaplan–Meier survival curves were generated using the KM Plotter online tool (http://kmplot.com), segregating BC patients for EMILIN1 transcript/HER2 ratio, using the most appropriate cut-off value in gene chip mRNA data from the TCGA dataset. *N* = 1879 BC patients for overall survival and *N* = 2765 for distant metastasis-free survival.

### Reporting summary

Further information on research design is available in the Nature Research Reporting Summary linked to this article.

### Supplementary information

Supplementary Information

Reporting summary

## Data Availability

All data supporting the findings of this study are available in the paper and its Supplementary Information. Any further information can be obtained from the corresponding author on reasonable request.
