# Supplementary material for: Loss of the extracellular matrix glycoprotein EMILIN1 accelerates Δ16HER2-driven breast cancer initiation in mice
Source: NPJ Breast Cancer. 2024 Jan 6;10:5. doi: 10.1038/s41523-023-00608-0 (PMC10771445; doi:10.1038/s41523-023-00608-0)

Supplementary Information

Loss of the extracellular matrix glycoprotein EMILIN-1 accelerates Δ16HER2-driven breast cancer initiation in mice

Andrea Favero[1], Ilenia Segatto[1], Alessandra Capuano[1], Maria Chiara Mattevi[1], Gian Luca Rampioni Vinciguerra[1,2], Lorena Musco[1], Sara D'Andrea[1], Alessandra Dall'Acqua[1], Chiara Gava[1], Tiziana Perin[3], Samuele Massarut[4], Cristina Marchini[5], Gustavo Baldassarre[1], Paola Spessotto[1] and Barbara Belletti[1*]

[1] *Unit of Molecular Oncology, Centro di Riferimento Oncologico (CRO) di Aviano, IRCCS, National Cancer Institute, 33081 Aviano, Italy.*

[2] *Faculty of Medicine and Psychology, Department of Clinical and Molecular Medicine, University of Rome "Sapienza", Santo Andrea Hospital, 00189 Rome, Italy.*

[3] *Unit of Pathology, Centro di Riferimento Oncologico (CRO) di Aviano, IRCCS, National Cancer Institute, 33081 Aviano, Italy.*

[4] *Unit of Breast Surgery, Centro di Riferimento Oncologico (CRO) di Aviano, IRCCS, National Cancer Institute, 33081 Aviano, Italy.*

[5] *School of Biosciences and Veterinary Medicine, Biology Division, University of Camerino, via Gentile III da Varano, 62032 Camerino, Italy.*

Supplementary Information contains Supplementary Table 1 and Supplementary Figures 1-4.

Supplementary Table 1

Gene	*Primer Forward 5' -> 3'*	*Primer Reverse 5' -> 3'*
Human D16HER2	CTGCACCCACTCCCCTCTGAC	CTGCCGTCGCTTGATGAGGATC
Human EMI1	CCAAAGCATCATGTACCG	ACTCTCAGCACAGTCATC
Human GAPDH	GAAGGTGAAGGTCGGAGTC	GAAGATGGTGATGGGATTTC
Murine ACC	CTGGCTGCATCCATTATGTCA	TGGTAGACTGCCCGTGTGAA
Murine ALDOA	CACGAGACACTGTACCAGAAGG	TTGTCTCGCCATTGGTTCCTGC
Murine ELF5	GTGGCATCAAGAGTCAAGACTGTC	CTCAGCTTCTCGTACGTCATCCTG
Murine EMI1	TAGCTGCTGCACCTTCTCTG	GGCAGCCATCTCAGTGGA
Murine ENO1	TACCGCCACATTGCTGACTTGG	GCTTGTTGCCAGCATGAGAACC
Murine ER	CTGGACAGGAATCAAGGTAA	AGAAACGTGTACACTCCGG
Murine FASN	ATTGGTGGTGTGGACATGGTC	CCCAGCCTTCCATCTCCTG
Murine FBP1	TGCTGAAGTCGTCCTACGCTAC	TTCCGATGGACACAAGGCAGTC
Murine G6PC	AGGTCGTGGCTGGAGTCTTGTC	GTAGCAGGTAGAATCCAAGCGC
Murine G6PDX	GACCAAGAAGCCTGGCATGTTC	AGACATCCAGGATGAGGCGTTC
Murine GAPDH	TGACCACAGTCCATGCCATC	GACGGACACATTGGGGGTAG
Murine GPI1	CCATCAAGGTGGACGGCAAAGA	CCGTGATGGATTTGCCAGTGTAC
Murine HK1	GAAAGGAGACCAACAGCAGAGC	TTCGTTCCTCCGAGATCCAAGG
Murine ITGA4	CAGGCATTCATGCGGAAAGAC	CTTCTGCTGAAGGATCGGCT
Murine ITGA9	CTGGAGCACTTCCACGACAA	ACCACGAGTCCTCCCTCG
Murine ITGB1	TTCAGACTTCCGCATTGGCT	AATGGGCTGGTGCAGTTTTG
Murine LDHA	ACGCAGACAAGGAGCAGTGGAA	ATGCTCTCAGCCAAGTCTGCCA
Murine LPL	CCAATGGAGGCACTTTCCA	TGGTCCACGTCTCCGAGTC
Murine PFKFB3	TCATCGAGTCGGTCTGTGACGA	CATGGCTTCTGCTGAGTTGCAG
Murine PFKM	CTGTTCGCTCTACCGTGAGGAT	TTGGAACCACCTTGACCAGTCC
Murine PGAM2	CTGGAATGAGGAGATCGCACCT	ATTCCAGTGGGCAGGTTCAGCT
Murine PGK1	GATGCTTTCCGAGCCTCACTGT	ACCAGCCTTCTGTGGCAGATTC
Murine PGM1	CAACGCACTGAAGGAGCTACTC	GGCACCAAGTTCTTCACAGAGG
Murine PgR Fw	TATGGCGTGCTTACCTGTGG	ACTTACGACCTCCAAGGAGGA
Murine PKM	CAGAGAAGGTCTTCCTGGCTCA	GCCACATCACTGCCTTCAGCAC
Murine PPARγ	TGTGGGGATAAAGCATCAGGC	CCGGCAGTTAAGATCACACCTAT
Murine Prl-R	TGAGGACGAGCGGCTAATG	GGTGTGTGGGTTTAACACCTTGA
Murine RANK-L	CAGCCGAGACTACGGCAAGT	AGGATCCATCTGCGCTCGAAA
Murine SLC2A1	GCTTCTCCAACTGGACCTCAAAC	ACGAGGAGCACCGTGAAGATGA
Murine SLC2A4	GGTGTGGTCAATACGGTCTTCAC	AGCAGAGCCACGGTCATCAAGA
Murine SREBP-1C	GGAGCCATGGATTGCACATTTG	CAAATAGGCCAGGGAAGTCAC
Murine TGF-β1	TGGAGCAACATGTGGAACTC	CAGCAGCCGGTTACCAAG
Murine TGF-β2	TCCCCTCCGAAAATGCCATC	ACTCTGCCTTCACCAGATTCG

Supplementary Figures

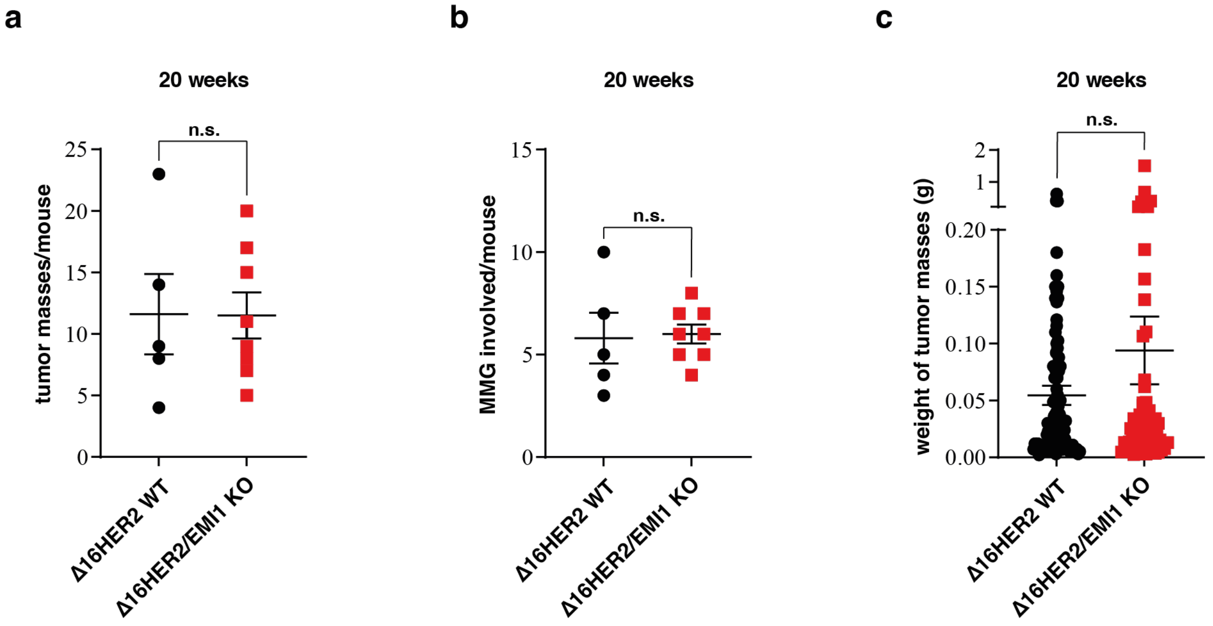

Supplementary Figure 1. Emilin1 loss does not impact on tumor multiplicity and tumor weight in Δ16HER2 mouse mammary glands. a, Graph reports the number of tumor masses/mouse collected from 20 weeks-old Δ16HER2 WT and Δ16HER2/EMI1 KO mice at the time of necroscopy. At least five animals/genotype were analyzed. **b**, Quantification of the number of murine mammary gland that displayed tumor formation in 20 weeks-old females. At least five animals/genotype were analyzed. **c**, Graph shows the weight of tumor masses collected from 20 weeks-old Δ16HER2 WT and Δ16HER2/EMI1 KO mice at the time of necroscopy. At least eight mice/genotype were evaluated and fifty-seven tumor masses were weighted for each genotype. Graphs report the mean ± SEM. Statistical significance was calculated using Student t test and indicated by a $P < 0.05$.

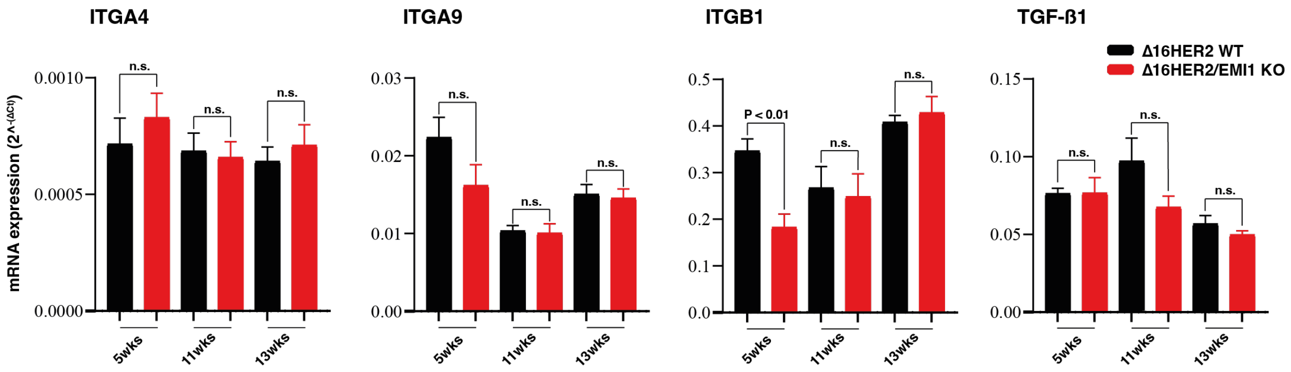

Supplementary Figure 2. α4β1 and α9β1 integrin receptors and TGFβ are expressed at low level on Δ16HER2 mouse mammary epithelial cells, both EMI1 WT and KO. From left to right, graphs report qRT-PCR evaluation of ITGA4, ITGA9, ITGB1 and TGFB transcripts in mouse mammary glands collected at 5, 11 and 13 weeks-of-age. At least three mice per genotype were analyzed. Graphs report the mean ± SEM. Statistical significance was calculated using Student t test and indicated by a $P < 0.05$.

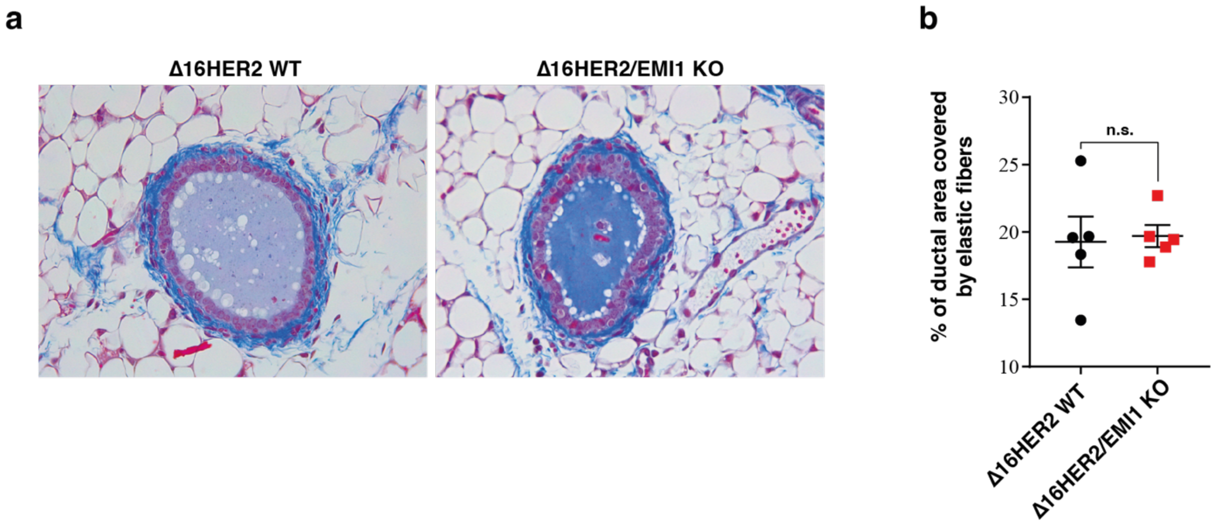

Supplementary Figure 3. Loss of Emilin1 does not alter the deposition of elastic fibers in Δ16HER2 mouse mammary gland. a, Masson's trichrome staining of mouse mammary gland. Both the third and the fourth mammary gland were evaluated and at least 5 fields for each slide were analyzed. Magnification 40X. **b**, Graph represents the percentage of the area covered by elastic fibers (blue dye) with respect to the total area of the duct, reported as the mean ± SEM. Statistical significance was calculated using Student t test and indicated by a $P < 0.05$.

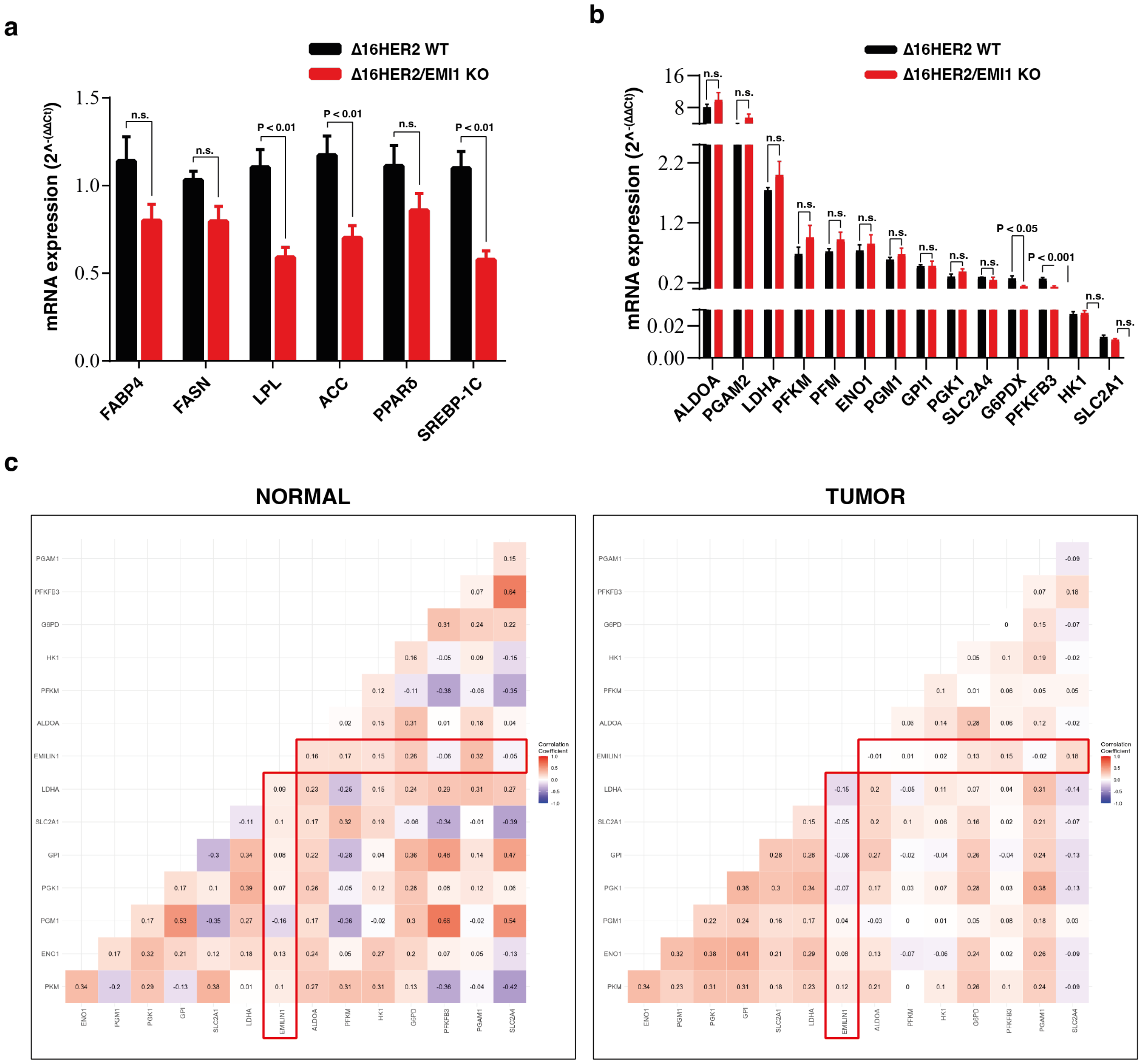

Supplementary Figure 4. Emilin1 KO does not modify fatty acid metabolism and glucose metabolism in Δ16HER2 mouse mammary gland. a, qRT-PCR evaluation of key regulators in the fatty acid metabolism. At least five mice per genotype were included in the analysis. **b**, qRT-PCR analysis of fundamental genes involved in glucose metabolism. At least three mice per genotype were analyzed. Graphs report the mean ± SEM. Statistical significance was calculated using Student t test and indicated by a $P < 0.05$. **c**, Graphs report the correlation between Emilin1 and glucose-related genes in healthy tissue (left) and breast tumor (right). Correlation data were retrieved from TNMplot, which collects RNA-seq data from TCGA-BRCA database and TARGET projects, comparing healthy tissues (n=403) and primary mammary tumors (n=1097). In all graphs, significance was calculated by Student t test and it is indicated by a $P < 0.05$.

Uncropped and unprocessed scans of the western blots

from Figure 3c

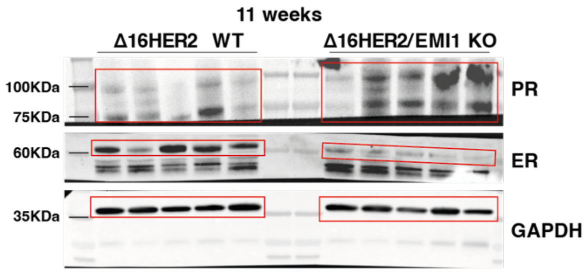

from Figure 4d

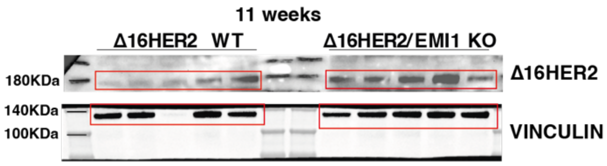

from Figure 4l

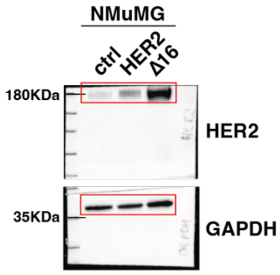

Supplement: Supplementary file 1 — Supplementary Information [file 41523_2023_608_MOESM1_ESM.pdf]
