# Supplementary material for: Loss of the extracellular matrix glycoprotein EMILIN1 accelerates Δ16HER2-driven breast cancer initiation in mice
Source: NPJ Breast Cancer. 2024 Jan 6;10:5. doi: 10.1038/s41523-023-00608-0 (PMC10771445; doi:10.1038/s41523-023-00608-0)
Supplement: Supplementary file 2 — Reporting summary [file 41523_2023_608_MOESM2_ESM.pdf]

nature portfolio

Corresponding author(s): Barbara Belletti

Last updated by author(s): Oct 17, 2023

Reporting Summary

Nature Portfolio wishes to improve the reproducibility of the work that we publish. This form provides structure for consistency and transparency in reporting. For further information on Nature Portfolio policies, see our Editorial Policies and the Editorial Policy Checklist.

Statistics

For all statistical analyses, confirm that the following items are present in the figure legend, table legend, main text, or Methods section.

n/a	Confirmed	
☐	☒	The exact sample size (*n*) for each experimental group/condition, given as a discrete number and unit of measurement
☐	☒	A statement on whether measurements were taken from distinct samples or whether the same sample was measured repeatedly
☐	☒	The statistical test(s) used AND whether they are one- or two-sided<br>*Only common tests should be described solely by name; describe more complex techniques in the Methods section.*
☒	☐	A description of all covariates tested
☒	☐	A description of any assumptions or corrections, such as tests of normality and adjustment for multiple comparisons
☐	☒	A full description of the statistical parameters including central tendency (e.g. means) or other basic estimates (e.g. regression coefficient) AND variation (e.g. standard deviation) or associated estimates of uncertainty (e.g. confidence intervals)
☒	☐	For null hypothesis testing, the test statistic (e.g. *F*, *t*, *r*) with confidence intervals, effect sizes, degrees of freedom and *P* value noted<br>*Give P values as exact values whenever suitable.*
☒	☐	For Bayesian analysis, information on the choice of priors and Markov chain Monte Carlo settings
☒	☐	For hierarchical and complex designs, identification of the appropriate level for tests and full reporting of outcomes
☐	☒	Estimates of effect sizes (e.g. Cohen's *d*, Pearson's *r*), indicating how they were calculated

Our web collection on statistics for biologists contains articles on many of the points above.

Software and code

Policy information about availability of computer code

Data collection	Data were collected using Excel software
Data analysis	GraphPad PRISM software (version 6.0) were used for statistical analysis

For manuscripts utilizing custom algorithms or software that are central to the research but not yet described in published literature, software must be made available to editors and reviewers. We strongly encourage code deposition in a community repository (e.g. GitHub). See the Nature Portfolio guidelines for submitting code & software for further information.

Data

Policy information about availability of data

All manuscripts must include a data availability statement. This statement should provide the following information, where applicable:

- Accession codes, unique identifiers, or web links for publicly available datasets
- A description of any restrictions on data availability
- For clinical datasets or third party data, please ensure that the statement adheres to our policy

All data supporting the findings of this study are available in the paper and its Supplementary Information.

Research involving human participants, their data, or biological material

Policy information about studies with human participants or human data. See also policy information about sex, gender (identity/presentation), and sexual orientation and race, ethnicity and racism.

Reporting on sex and gender	We used breast cancer samples from females
Reporting on race, ethnicity, or other socially relevant groupings	No race, ethnicity, or other socially relevant groupings criteria were used
Population characteristics	NA
Recruitment	Patients were enrolled in the study upon signing an informed consent
Ethics oversight	The study was approved by the Institutional Research Board (IRB-06-2017) of the Centro di Riferimento Oncologico (CRO) di Aviano (Italy).

Note that full information on the approval of the study protocol must also be provided in the manuscript.

Field-specific reporting

Please select the one below that is the best fit for your research. If you are not sure, read the appropriate sections before making your selection.

☒ Life sciences ☐ Behavioural & social sciences ☐ Ecological, evolutionary & environmental sciences

For a reference copy of the document with all sections, see nature.com/documents/nr-reporting-summary-flat.pdf

Life sciences study design

All studies must disclose on these points even when the disclosure is negative.

Sample size	No sample size calculation was pre evaluated. For in vitro experiments at least three biological replicates were used according to good laboratory practice.<br>For in vivo experiments the number of animals needed was calculated through an ANOVA with 3 dependent variables (parameters), and the expected effect f2(V). For each experiment, the a priori error probabilities were set α=0.05 and β=0.20.<br>Our experimental design was carefully calibrated to balance, on the one hand, the need to reduce the number of animals used to a minimum (in compliance with the 3R principles) and, on the other, the need to obtain statistically significant results from experimentation.
Data exclusions	No sample/animal was excluded from the study. No a priori criteria were established for the human samples.
Replication	All in vitro data represent the mean of at least three biological replicates and/or three indepdent experiments
Randomization	Animals were assigned to each group depending on their genotype
Blinding	Pathological analyses were performed in blind by expert pathologists

Reporting for specific materials, systems and methods

We require information from authors about some types of materials, experimental systems and methods used in many studies. Here, indicate whether each material, system or method listed is relevant to your study. If you are not sure if a list item applies to your research, read the appropriate section before selecting a response.

Materials & experimental systems

n/a	Involved in the study	
☐	☒	Antibodies
☐	☒	Eukaryotic cell lines
☒	☐	Palaeontology and archaeology
☐	☒	Animals and other organisms
☐	☒	Clinical data
☒	☐	Dual use research of concern
☒	☐	Plants

Methods

n/a	Involved in the study	
☒	☐	ChIP-seq
☒	☐	Flow cytometry
☒	☐	MRI-based neuroimaging

Antibodies

Antibodies used	HER2 (Abcam, #ab134182), Ki67 (Abcam, #ab15580); EMILIN-1 (rabbit polyclonal As556, home-made); PgR (Thermofisher, PA5-16440); ER (Thermofisher, MA1-411); GAPDH (Cell Signalling, #5174); Vinculin (Santa Cruz, sc7649, N19); horseradish peroxidase-conjugated secondary antibodies (GE Healthcare); AlexaFluor® 488- or 568- -conjugated (Invitrogen, 1:200)
Validation	Commercially available Abs were validated by following the manufacturer's protocol. EMILIN1 antibody was validated using tissues fro EMIKO animals as negative controls.

Eukaryotic cell lines

Policy information about cell lines and Sex and Gender in Research

Cell line source(s)	NMuMG cells were a kind gift of Dr Andrei V. Bakin (Roswell Park Comprehensive Cancer Center, Buffalo, NY)
Authentication	Commecially available cell were authenticated according to the PowerPlex® 16 HS System protocol and using Genemapper ID Ver 3.2.1. Authenticated cells were kept frozen and used no later than 2 months in culture for performing the experiments.
Mycoplasma contamination	Mycoplasma contamination was assessed at least once a month using the MycoAlert test (Lonza).
Commonly misidentified lines (See ICLAC register)	Not applicable

Animals and other research organisms

Policy information about studies involving animals; ARRIVE guidelines recommended for reporting animal research, and Sex and Gender in Research

Laboratory animals	Mus Musculus, FVB MMTV-Δ16HER2 transgenic mouse model; FVB EMILIN KO mouse model
Wild animals	Not applicable
Reporting on sex	Females only
Field-collected samples	Not applicable
Ethics oversight	Animal experimentation was reviewed and approved by Centro di Riferimento Oncologico di Aviano (CRO) Institutional Organism for Animal Wellbeing (OPBA) and by the Italian Ministry of Health (aut. No. 616/2015-PR and 630/2020-PR, released to B.B.).

Note that full information on the approval of the study protocol must also be provided in the manuscript.

Clinical data

Policy information about clinical studies

All manuscripts should comply with the ICMJE guidelines for publication of clinical research and a completed CONSORT checklist must be included with all submissions.

Clinical trial registration	Not applicable
Study protocol	Biospecimens were obtained from patients who gave their informed consent, under protocols approved on 15.11.2018 by the Institutional Research Board (IRB-06-2017) of the Centro di Riferimento Oncologico (CRO) di Aviano, Italy.
Data collection	Described in methods section
Outcomes	Not applicable

Plants

Seed stocks	Not applicable
Novel plant genotypes	Not applicable
Authentication	Not applicable
